# Quantifying Chaos by Various Computational Methods. Part 2: Vibrations of the Bernoulli–Euler Beam Subjected to Periodic and Colored Noise

**DOI:** 10.3390/e20030170

**Published:** 2018-03-05

**Authors:** Jan Awrejcewicz, Anton V. Krysko, Nikolay P. Erofeev, Vitalyi Dobriyan, Marina A. Barulina, Vadim A. Krysko

**Affiliations:** 1 Department of Automation, Biomechanics and Mechatronics, Lodz University of Technology, 1/15 Stefanowski St., 90-924 Lodz, Poland; 2 Cybernetic Institute, National Research Tomsk Polytechnic University, 30 Lenin Avenue, 634050 Tomsk, Russia; 3 Department of Applied Mathematics and Systems Analysis, Saratov State Technical University, 77 Politechnicheskaya, 410054 Saratov, Russia; 4 Department of Mathematics and Modeling, Saratov State Technical University, 77 Politechnicheskaya, 410054 Saratov, Russia; 5 Precision Mechanics and Control Institute, Russian Academy of Science, 24 Rabochaya Str., 410028 Saratov, Russia

**Keywords:** geometric nonlinearity, Bernoulli–Euler beam, colored noise, noise induced transitions, true chaos, Lyapunov exponents, wavelets

## Abstract

In this part of the paper, the theory of nonlinear dynamics of flexible Euler–Bernoulli beams (the kinematic model of the first-order approximation) under transverse harmonic load and colored noise has been proposed. It has been shown that the introduced concept of phase transition allows for further generalization of the problem. The concept has been extended to a so-called noise-induced transition, which is a novel transition type exhibited by nonequilibrium systems embedded in a stochastic fluctuated medium, the properties of which depend on time and are influenced by external noise. Colored noise excitation of a structural system treated as a system with an infinite number of degrees of freedom has been studied.

## 1. Introduction

There are some well-recognized and thoroughly investigated scenarios of transition from regular to chaotic vibration of deterministic systems, such as the well-known Ruelle–Takens scenario, in which the key role is played by only three frequencies [1]. It was found that the “noisy” behavior was exhibited by a “strange attractor” occurred after three successive limit cycle bifurcations. In addition, a period doubling bifurcation route to chaos as well as a scenario of transition by means of intermittency were also detected and studied [2]. However, a stochastic character of the matter may induce more interesting regimes of nonlinear vibrations than that reported in the available literature [3]. Transitions from one nonlinear state to another are analogous to equilibrium phase transitions and they are met in nonequilibrium processes under deterministic external inputs. A question arises: Is it possible to extend the classical transitions to processes in which a key role is played by noise? It implies theoretical investigations of the transitions induced by noise based on the numerical study. The investigation of deterministic chaotic multi-dimensional systems has been carried out (e.g., [4,5,6,7,8,9]). The aim of the present work was to extend the mentioned approaches to the case of the noise-induced transitions into chaos.

The investigation of complex vibrations of continuous systems belongs to challenging problems of modern dynamics. In the works of Amabili [10,11], distributed mechanical systems have been considered in the form of a shallow shell of double curvature and a closed cylindrical shell with 16 degrees of freedom. The problem has been solved by Galerkin’s method, but the number of similar works is limited. In most of the available papers, reduced-model approaches have been used to simplify these considerations. Namely, one can find numerous papers attempting to significantly simplify the studied problem of an infinite dimension to one- or two-degree-of-freedom reduced models. The obtained results are usually validated under strong assumptions and can be treated rather as qualitative since an increase in the number of modes often yields essentially different outcome.

One of the most important problems encountered when studying chaotic vibrations is the reliability of the obtained results in the case of true/real chaos. True/real chaos means a reliable and validated chaotic solution obtained numerically, and is contrary to a possible “false chaotic solution” caused by the errors associated with the used numerical algorithms as well as time and spatial steps. A more detailed description can be found in Reference [12]. The majority of investigations rely on numerical simulations, which may accumulate the errors yielding false results regarding the estimation of true chaos. Therefore, one of the aims of the present paper was to validate the truth of chaotic orbits by different numerical methods, since the obtained results may depend on the employed numerical method.

Proper modeling and the way of solving the spatiotemporal nonlinear dynamics of Euler–Bernoulli beams and other structural members governed by nonlinear PDEs plays a crucial role in obtaining true/reliable results. The majority of the research devoted to dynamics of continuous mechanical systems has its origin in engineering and, depending on the needs, the governing PDEs should satisfy different requirements regarding the results accuracy and duration of the computations. For instance, in static problems, it is rather expected to get highly accurate models, which usually require long computational time since the commonly used program packages are based on the FEM (finite element method) and/or the FDM (finite difference method) as well as the higher-order Galerkin methods, the Ritz-type methods, etc. Contrarily, dynamic problems (often) require control, which means from the point of view of complexity of the governing PDEs that the developed model should be rather dedicated to the considered task and the nonlinear PDEs should be appropriately reduced to get reliable and relatively fast numerical results. In general, there is no way to solve the infinite-dimension nonlinear problems analytically.

Thus, the knowledge and experience about proper modeling of nonlinear vibrations of structural members as well as the way of getting solution to the governing PDEs with the supplemented boundary and initial conditions are the key factors in achieving reliable results.

To exhibit the importance of the mentioned problem, we used the classical Lorentz [13] results, showing that, depending on the time step, the used numerical schemes (Euler and Runge–Kutta) and Taylor series truncation, one can get the so-called “computational chaos”, i.e., a spurious solution not validated by either real laboratory experiment or qualitatively different numerical approaches. As reported by Yee and Sweby [14], even small time steps and the employment of the classical Euler, Runge–Kutta, predictor-corrector schemes may yield spurious solutions (sometimes coexisting with true solutions). It may also happen that the decrease in the time step may yield worse computational results. For instance, Corless et al. [15] pointed out that a true/correct chaotic orbit can be even suppressed.

Note that the investigation of spatial dynamics of structures involves distribution in time and space and, usually, the problem should be reduced to that of many nonlinear ODEs and possibly AEs (algebraic equations). However, the first-mode approximations are usually used to investigate chaotic vibrations of simple 1D structural members (beams). As mentioned above, this approach requires to be rather strictly realized in real/engineering problems (see, e.g., [16,17,18,19]).

In the following, the description of numerous works devoted to study nonlinear beams, including the Euler–Bernoulli beams, is omitted and we refer only to the recent tendency in analyzing nonlinear dynamics of beams taking into account a stochastic/random excitation.

Bar-Yoseph et al. [20] employed the space-time spectral element method for a nonlinear Euler–Bernoulli beam using a generalized Galerkin approach as well as a discontinuous mixed Galerkin method for the temporal discretization. However, they considered only the two first modes.

Based on short datadets, Dunne and Ghanbari [21] predicted extreme exceedance probabilities matched with experimental investigations of highly nonlinear clamped-clamped beam vibrations driven by band-limited white noise.

Ge and Xu [22] proposed a stochastic nonlinear dynamical model of vibrations of a flexible beam under axial excitation, considering random environmental factors. The governing PDEs were simplified using the stochastic average theory, and then the methods of Lyapunov exponents and boundary classification with the account of diffusion process were employed to study the stochastic stability of the trivial solutions. Then, the stochastic Hopf bifurcation was studied. However, the problem was limited to investigating only one second-order ODE.

Cacciola et al. [23] analyzed the vibrational response of a beam with an edge nonpropagating crack by means of a stochastic analysis to detect the presence and the location of structural damage. Numerical investigation was used to show that a cantilever beam exhibited high sensitivity of the skewness coefficient of the rotational degrees of freedom. However, only third- and fourth-order statistical quantities of the response were evaluated, and the obtained results were not validated experimentally.

Sahoo et al. [24] studied nonlinear transverse vibration of a simply-supported traveling Euler–Bernoulli beam under principal parametric resonance and in presence of internal resonance. However, the problem was essentially limited to studying only the principal parametric resonance of the first mode by employing the method of multiple scales.

Lan and Qin [25] investigated the energy harvesting from the horizontal coherent resonance of a vertical cantilever beam subjected to the vertical base excitation. They demonstrated the horizontal coherence resonance when the beam was excited by a vertical white noise. However, derivation of the governing PDEs was not shown, but only the experimental results were reported.

Xu and Ma [26] investigated, in a rigorous mathematical way, the existence of a compact random attractor for the floating beam equation with strong damping and white noise. However, their results seem to be mathematically-oriented. The authors did not present any numerical results, and hence direct application of the obtained results to real problems is rather limited.

It is known and commonly accepted that the main characteristic feature of deterministic chaos is its essential dependence on the initial conditions. The definition of chaos, initially formulated by Devaney in 1989 [27], consists of three parts. In addition to the essential dependence on the initial conditions, there is a chaotic mixing condition referred to as the transitivity and regularity condition, measured by a density of periodic points. On the other hand, in 1992, Banks et al. [28] proved that the transitivity and regularity conditions imply the sensitivity to the initial conditions.

A more rigorous chaos definition, given by Knudsen [29] also exists, where a function defined on a bounded metric space is chaotic if it has a dense orbit and exhibits essential dependence on the initial conditions.

Owing to chaos definition proposed by Gulick [12], chaos exists if either there is an essential dependence on the initial conditions or a chaotic function has a positive Lyapunov exponent in its each point, and hence if it finally does not tend to a periodic or quasi-periodic orbit.

In this paper, similar to in Part 1, we employed chaos definition given by Gulick [12], i.e., we validated true chaotic solutions by employing computations of the Lyapunov exponents. The obtained solutions depend on the chosen kinematic hypothesis, boundary and initial conditions, number of beam partitions in the case of the FDM (finite difference method), the chosen method of solutions to the Cauchy problems, and the chosen time step.

To detect true chaotic vibrations when numerically solving the problem devoted to nonlinear vibrations of a package consisting of two beams with a clearance and subjected to transversal sinusoidal load and the colored noise, the following complex investigations were employed:

1. Since PDEs are reduced to ODEs (Cauchy problem) using the FDM of second-order accuracy, their solution essentially depends on the number of beam length partitions (nodes). We need to find a number of partitions *n*, for which the solution coincided with the case of using *n* + 1 partitions. Furthermore, we want to achieve convergence even with respect to time history in the case of chaotic orbits. In monograph [30] dealing with a similar problem, the convergence was achieved only with respect to the periodic vibrations, and in the case of chaotic vibrations, only integral convergence was accepted, i.e., coincidence of power spectra was used to validate true chaos.
2. The Cauchy problem was solved numerically and it is known that it depends on the chosen method and the integration step, which is why we chose different methods to validate the computational results: fourth order the Runge–Kutta method (RK4) and the second order Runge–Kutta method (RK2) [31], the fourth order Runge–Kutta–Fehlberg method (RKF45) [32,33], the fourth order Cash–Karp method (RKCK) [34], the eighth order Runge–Kutta–Prince–Dormand method (RK8PD) [35], the implicit second order Runge–Kutta method (rk2imp), and the fourth order Runge–Kutta implicit method (rk4imp). The implicit method makes it possible to include an arbitrary form of the used matrix of the related coefficients, whereas the RKF45, RKCK, and RK8PD methods allow for the automatic change of the computational step as well as to control errors introduced by integration.
3. For each of the introduced numbers of partitions of the beam length and the solutions to the Cauchy problems, the time histories (vibration signals), 2D and 3D phase portraits, the Fourier power spectra, the Morlet wavelets, snapshots of beam deflections, and Poincaré maps were constructed. For the chosen signal, a 2D wavelet spectrum was also constructed. The following mother wavelets were employed: Haar [36]; Shannon–Kotelnikov and Meyer [37]; Daubechies wavelets from db2 up to db16 [38]; coiflets, simlets, and the Morlet and complex Morlet wavelets [39]; and the wavelets based on the derivative of the Gauss function of the order higher than eight. However, the Haar and Shannon–Kotelnikov wavelets were unsuitable for our purpose. Namely, the first one was badly localized with respect to frequency, and the second one with respect to time.

The analysis of the wavelet spectra yielded by the Daubechies wavelets, coiflets and simlets showed that an increase in the filter order results in an increase in the frequency deflection/localization. The results obtained by the Daubechies wavelets, simlets and coiflets were practically the same, which validates them. However, their frequency localization was not enough suitable to be reliable while studying the character of vibrations of the studied continuous mechanical system.

As expected, the results obtained based on the derivative of the Gauss functions yielded an increase in the frequency resolution with an increase in the derivative order. The power spectra obtained based on the Meyer wavelet [40,41] (a smoothened variant of the Shannon–Kotelnikov wavelet) were better localized in the low-frequency band than the Morlet power spectra. However, the latter ones fit the higher part of the frequency spectrum better. In the following, we give the results based on the mother Morlet wavelets. In Morlet wavelets, there is a lack of scaling function φ, the function ψ does not have a compact carrier and it is given explicitly. The complex Morlet and Gauss wavelets exhibit better localization with respect to frequency, but their real counterpart is better localized in time. Therefore, to study complex vibrations of dynamical continuous systems (beams), one can use both real and complex Morlet wavelets based on the derivatives of the Gauss functions of the order higher than sixteen.

4. Since we employed the Gulick [12] definition of chaos, we needed to compute and validate signs of the largest Lyapunov exponents (LLEs). Spectra of Lyapunov exponents were estimated based on the Kantz [42], Wolf [43] and Rosenstein [44] methods. The results were eventually accepted if they agreed to the fourth decimal place.

Vibrations of geometrically nonlinear Bernoulli–Euler beam (kinematic model of the first approximation) were studied as a system with many degrees of freedom subjected to colored noise. White noise states for a general stationary probabilistic process X(t) with a constant spectral density. The term “white” corresponds to the white light, the spectrum of which combines the whole palette of colors in the visible spectrum part. The correlation white noise function is governed by the formula $B(t)=\sigma^{2}\delta (t),$ where $\sigma^{2}$ is a constant, and δ(t) stands for the delta function. An essentially nonlinear character of the behavior of plates and shells under loading by noise pressure has been found in several works. The Gaussian white noise model is very useful and appropriate to study different natural processes. The colored noise spectrum corresponds to the distribution of the visible part of the light spectrum.

## 2. Problem Statement and the Mathematical Model

**Figure 1:**
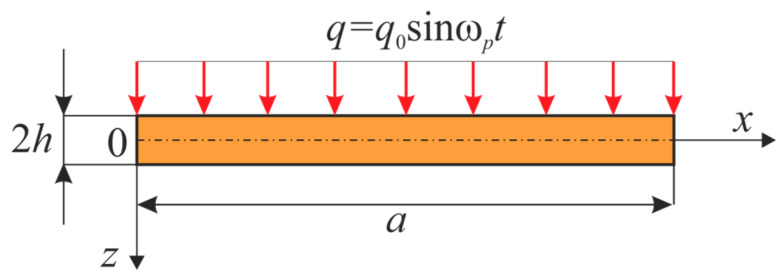
Scheme of the investigated beam.

We considered a one-layer isotropic elastic beam (Figure 1) representing a 2D area of the space $R^{2}$ with the attached coordinate system introduced in the following way: in the beam body, a so-called reference line, also called a middle line, z=0 was fixed; the axis *OX* was directed from the left to the right along the middle line. The beam area Ω, in the given coordinate system, was defined as follows: Ω={x∈[0;a];−h≤z≤h}, 0≤t≤∞. The following notation was employed: 2h—beam height, a—beam length.

The mathematical model was constructed based on the Bernoulli–Euler hypothesis considering a nonlinear relation between deformations and displacements in the von Kármán form. The governing PDEs in displacements had the following form [45]:

(1) $$\begin{array}{l} E2h\left\{\frac{\partial^{2}u}{\partial x^{2}}+L_{3}(w,w)\right\}-2h\frac{\gamma }{g}\frac{\partial^{2}u}{\partial t^{2}}=0,\\ E2h\left\{L_{1}(u,w)+L_{2}(w,w)-{\left(2h\right)}^{2}\left(\frac{1}{12}\right)\frac{\partial^{4}w}{\partial x^{4}}\right\}+q+\dot{q}-2h\frac{\gamma }{g}\frac{\partial^{2}w}{\partial t^{2}}-2h\varepsilon \frac{\gamma }{g}\frac{\partial w}{\partial t}=0,\\ L_{1}(u,w)=\frac{\partial^{2}u}{\partial x^{2}}\frac{\partial w}{\partial x}+\frac{\partial u}{\partial x}\frac{\partial^{2}w}{\partial x^{2}},\;L_{2}(w,w)=\frac{3}{2}\frac{\partial^{2}w}{\partial x^{2}}{\left(\frac{\partial w}{\partial x}\right)}^{2},\;L_{3}(w,w)=\frac{\partial^{2}w}{\partial x^{2}}\frac{\partial w}{\partial x}, \end{array}$$

where: w(x,t) is the beam deflection, u(x,t) is the displacement along the OX axis, ε is the dissipation coefficient, q=q(x,t) is the transverse load, $\dot{q}(t)$ is the colored noise, E is Young’s modulus, *γ* is the specific volume beam weight, and g is the gravity of Earth.

The following non-dimensional quantities were introduced:

(2) $$\begin{array}{l} \bar{w}=\frac{w}{2h},\;\bar{u}=\frac{ua}{{\left(2h\right)}^{2}},\;\bar{x}=\frac{x}{a},\;\lambda =\frac{a}{2h},\;\bar{q}=q\frac{a^{4}}{{\left(2h\right)}^{4}E},\\ \bar{t}=\frac{t}{\tau },\;\tau =\frac{a}{c},\;c=\sqrt{\frac{Eg}{\gamma }},\;\bar{\varepsilon }=\varepsilon \frac{a}{c}. \end{array}$$

The system in Equation (1), taking into account Equation (2), took the following simple counterpart form

(3) $$\begin{array}{l} \frac{\partial^{2}u}{\partial x^{2}}+L_{3}\left(w,w\right)-\frac{\partial^{2}u}{\partial t^{2}}=0,\\ \frac{1}{\lambda^{2}}\left\{L_{2}(w,w)+L_{1}\left(u,w\right)-\left(\frac{1}{12}\right)\frac{\partial^{4}w}{\partial x^{4}}\right\}-\frac{\partial^{2}w}{\partial t^{2}}-\varepsilon \frac{\partial w}{\partial t}+q+\dot{q}=0, \end{array}$$

(bars over non-dimensional quantities were omitted).

Equation (3) was supplemented by the following boundary conditions: simple support

(4) $$w(0,t)=w(1,t)=u(0,t)=u(1,t)=\partial^{2}w(0,t)/\partial x^{2}=\partial^{2}w(1,t)/\partial x^{2}=0$$

or clamping

(5) w(0,t)=w(1,t)=u(0,t)=u(1,t)=∂w(0,t)/∂x=∂w(1,t)/∂x=0

on both beam ends.

In the case of mixed boundary conditions (clamping and simple support):

(6) $$w(0,t)=w(1,t)=u(0,t)=u(1,t)=\partial w(0,t)/\partial x={\partial^{2}w(1,t)/\partial x}^{2}=0$$

The initial conditions were as follows:

(7) $${w(x,t)|}_{t=0}={\frac{\partial w(x,t)}{\partial t}|}_{t=0}={u(x,t)|}_{t=0}={\frac{\partial u(x,t)}{\partial t}|}_{t=0}=0$$

## 3. Methods of Solution

### 3.1. FDM Method 

Wes substituted the differential operators with respect to x in Equations (3)–(7) by difference operators for the function w(x,t), u(x,t) with the help of the FDM with approximation $O\left(h^{2}\right)$. PDEs in Equation (3) were reduced to the following ODEs

(8) $${\ddot{w}}_{i}+\varepsilon_{1}{\dot{w}}_{i}=L_{1,h}(w_{i},u_{i}),\;{\ddot{u}}_{i}=L_{2,h}(w_{i},u_{i}),\;i=0,\ldots ,n,$$

where *n* is the number of partitions along *x*, *c* stands for the spatial step, and the difference operators *L*_1*,c*_ and *L*_2*,c*_ have the following form:

$$\begin{array}{l} L_{1,c}(w_{i},u_{i})=\frac{1}{\lambda^{2}}(-\frac{1}{12}\frac{1}{c^{4}}\left(w_{i+2}-4w_{i+1}+6w_{i}-4w_{i-1}+w_{i-2}\right)+\frac{1}{2c}(w_{i+1}-w_{i-1})\\ \times \frac{1}{{с}^{2}}\left(u_{i+1}-2u_{i}+u_{i-1}\right)+\frac{1}{2c}(w_{i+1}-w_{i-1})\frac{1}{{с}^{2}}\left(u_{i+1}-2u_{i}+u_{i-1}\right)+{\left(\frac{1}{2c}(w_{i+1}-w_{i-1})\right)}^{2}\frac{1}{c^{2}}\left(w_{i+1}-2w_{i}+w_{i-1}\right)+\\ \frac{1}{c^{2}}(w_{i+1}-2w_{i}+w_{i-1})\left(\frac{1}{2с}\left(u_{i+1}-u_{i-1}\right)+\frac{1}{8{с}^{2}}(w_{i+1}-w_{i-1})(w_{i+1}-w_{i-1})\right)),\\ \;L_{2,c}\left(w_{i},u_{i}\right)=\frac{1}{c^{2}}\left(u_{i+1}-2u_{i}+u_{i-1}\right)+\frac{1}{2c}\left(w_{i+1}-w_{i-1}\right)\frac{1}{c^{2}}\left(w_{i+1}-2w_{i}+w_{i-1}\right) \end{array}$$

In Equations (8), it is necessary to consider the values at the boundary points, which are defined by the boundary conditions. For the boundary conditions in Equation (4): $w_{-i}=-w_{i},$ $w_{0}=0,\;w_{n}=0,\;u_{0}=0,\;u_{n}=0$ (simple support). For the boundary conditions in Equation (5): $w_{-i}=-w_{i},$ $w_{0}=0,\;w_{n}=0,\;u_{0}=0,\;u_{n}=0$ (clamping). 

The initial conditions in Equation (7) can be rewritten in the difference form as follows:

$$w\left(x_{i}\right){|}_{t=0}=0;\;u\left(x_{i}\right){|}_{t=0}=0;\;\dot{w}\left(x_{i}\right){|}_{t=0}=0;\;\dot{u}\left(x_{i}\right){|}_{t=0}=0,\;\left(i=0,\ldots ,n\right).$$

### 3.2. Lyapunov Exponents

Wolf et al. [43] proposed an algorithm allowing for estimation of positive Lyapunov exponents based on the time series. It was shown that Lyapunov exponents correspond to either exponential fast divergence or convergence of the neighborhood orbits in the studied phase space. The employed concept of computation comes from the earlier method applied only to analytically defined mathematical models. The method traces a long term increase in a small volume chosen in the studied attractor and yields the largest Lyapunov exponent (LLE) based on one time series. The method does not require any knowledge about the evolutionary equations and phase coordinates. The algorithm for computing the LLE was described in Part 1 of our paper. As a reminder, the Kantz [42] and Rosenstein [44] methods are based on constructing the time-dependent function and take into account divergence of the nearby trajectories in the phase space in a certain time period. Then, they try to find the straightest part of this function (which is not always possible). A tangent of the inclination angle of this straight line yields the LLE.

### 3.3. Case Studies and Results Analysis

**Figure 2:**
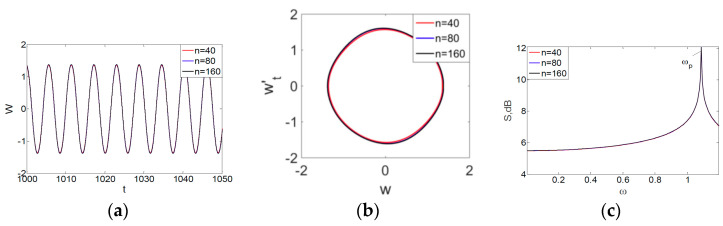
Convergence of the FDM (finite difference method) with respect to the vibrations signal and Fourier power spectrum (periodic orbits). (**а**) Signal (*n* = 40; 80; 160); (**b**) Phase portraits (*n* = 40; 80; 160); (**c**) Fourier power spectrum (*n* = 40; 80; 160).

**Figure 3:**
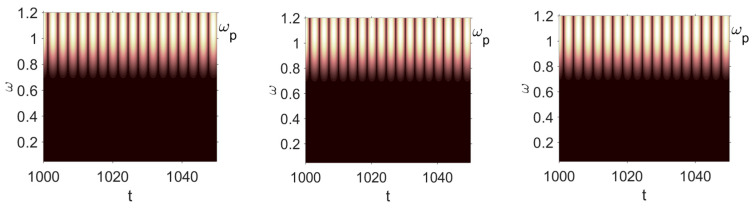
Convergence of the FDM with respect to the Morlet wavelet spectrum (periodic orbits).

Owing to the above-described methodology, we began with investigating the convergence of the FDM with approximations $О\left(h^{2}\right)$ and the Runge–Kutta methods as well as with comparing the results obtained by different Runge–Kutta methods. The comparisons were made by taking n=40;80;120;160;200; 240;280;320;360;400;440 nodes in the FDM when transiting PDEs in Equation (3), the boundary conditions in Equation (5) and the initial conditions in Equation (7) to ODEs (Cauchy problem). The Cauchy problem was solved by the Runge–Kutta methods of the second, fourth and eighth orders (Figure 2b reports the results obtained for n=360 yielded by different kinds of Runge–Kutta methods). The most reliable results were obtained for the eighth order Runge–Kutta method in the Prince–Dormand modified version. Convergence of the vibration periodic and chaotic signals was investigated. Figure 2 and Figure 3 present time histories/signals (Figure 2a), phase portraits (Figure 2b), power spectra (Figure 2c) and the Morlet wavelet spectra (Figure 3) for the periodic vibrations depending on the nodes number *n* = 40; 80; 160. Convergence of FDM was fully achieved for *n* = 80. All the mentioned vibration characteristics fully coincided.

**Figure 4:**
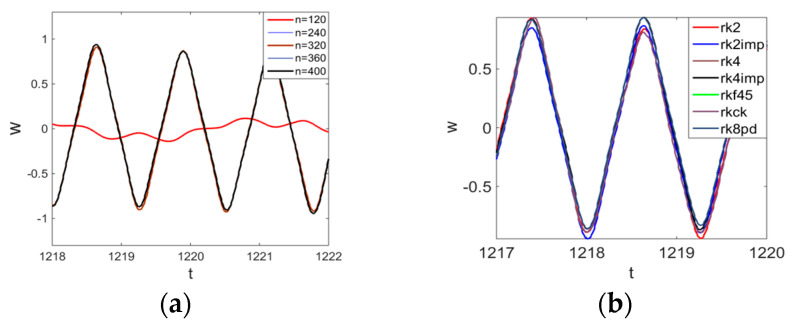
Signals obtained for n=120;240; 320; 360;400; (**a**) Convergence of *w*(0.5; *t*), *t* ∈ [1218; 1222] vs. Number of Nodes; (**b**) Convergence of *w*(0.5; *t*), *t* ∈ [1217; 1220] vs. Numerical Method Types.

Then, we studied convergence of the FDM for chaotic vibrations. Figure 4a presents the vibration signals w(0.5, t) obtained for n=120;240; 320; 360;400. For n=120, the deflection essentially differed from the deflection obtained for n=240;320;360;400 (for which the deflections coincided).

Owing to Point 3 of the described methodology, power spectra, phase portraits, and Morlet wavelet spectra for ω∈[0.2;5] are reported in Table 1. These characteristics were considered for n=120; 320;360; 400. In the power spectrum, for n=120, the independent frequencies $\omega_{1},\;\omega_{3},\;\omega_{5},\;\omega_{6},\;\omega_{8},\;\omega_{9},\;\omega_{10},\;\omega_{11},\;\omega_{12},\;\omega_{13},\;\omega_{15}$; the dependent frequencies $\omega_{14}=\frac{2}{5}\omega_{p},\;\omega_{7}=\frac{3}{5}\omega_{p}$; and a chaotic component were detected. An increase in the number of nodes changed the signal frequency spectrum (Fourier power spectrum). Eleven frequencies were detected for n=320 and twelve for n=360; 400. The phase portraits present washed out clouds converging to a ring with the increase in the number of nodes up to n=320; 360; 400. For n=120, the wavelet spectrum exhibits three frequencies $\omega_{p}$, $\omega_{3}$ and $\omega_{14}$, and an increase in the number of nodes yielded only two frequencies—$\omega_{p}$ and $\omega_{3}$. It means that convergence with respect to power spectra, phase portraits and wavelet spectra was achieved.

All Lyapunov exponents (Table 2) obtained by three mentioned methods are positive, and each of the methods is convergent with respect to n (a/(2 *h*) = 50).

To study behavior of nonlinear beams under harmonic $q=q_{0}\sin(\omega_{p}t)$ load and colored noise of amplitude $C_{n},$ a package of programs allowing for construction and analysis of the power spectra and Lyapunov exponents was developed. Beam vibrations were traced on the time interval t∈[0; 2024], the number of partitions in the FDM was n={80;400}, and the ratio of the beam length to the beam thickness was λ=50. All results were obtained for the beam center. Vibration signals, Fourier power spectra, Morlet wavelet-spectra, phase and modal portraits, autocorrelation functions and LLEs were analyzed for the given type of colored noise.

When carrying out the numerical study, we chose a frequency such that the addition of low-amplitude noise did not change the system state. This motivated us to consider the noise amplitude $C_{n}=5，000$ being compatible with the amplitude of periodic excitation.

The spectrum density of the employed colored noises is proportional to $1/f^{\alpha }$, where f stands for the frequency of the spectrum, and α takes the following values: α = −2—purple noise; α = −1—blue noise; α = 0—white noise; α = 1—pink noise; and α = 2—brown noise.

The values for all time series for colored noise were found in the interval [−1; 1] and then multiplied by $C_{n}$ and added to the sinusoidal load.

Table 3 includes Morlet wavelet transform, Fourier spectrum, vibrational signal, Poincaré map, and 2D phase portraits for the beam partitions *n* = {80; 400} (for fixed $C_{n}$ = 5000). Results presented in Table 4 show that the most “noisy parts” of the Fourier and wavelet spectra were yielded by pink noise with dominating low-frequency band. The wavelet spectrum and the Fourier spectrum exhibit chaos on for all frequencies, phase portraits present dense clouds of phase plots, whereas Poincaré maps exhibit “chaotically” distributed points. Spectral densities of blue and white noise are similar, but white noise exhibits more low-frequency power, and hence the system is less chaotic in this case. On the contrary to the Fourier spectra, the phase portraits for white and brown noise are very similar. The strength of chaos again depends on low-frequency components of noise. This is validated by purple noise with dominating high-frequency components.

The methodology for finding the largest Lyapunov exponents by the Kantz and Rosenstein methods required the estimation of a tangent of the inclination angle of the most linear part of the time-dependent functions (Table 5). For this purpose, the least-squares algorithm was employed.

All possible points of the graph are taken for approximation at a given minimum length in time in percentage (in this case 30%) and the interval corresponding to the smallest sum of squares of the deviation of the initial data from this interval is selected.

The function *S*(*τ*) represents the dependence of the average divergence of the corresponding trajectories for the initial states governed by the inequality $\left|x\left(t\right)-x\left(t_{j}\right)\right|<\varepsilon$ (ε is a certain small quantity) for the considered time evolution *τ*.

## 4. Concluding Remarks

The second part of this paper dealt with more complicated nonlinear dynamics exhibited by the Bernoulli–Euler beam subjected to deterministic and noisy input. In this case, the following main conclusions were yielded.

1. Convergence of the employed numerical methods was investigated with respect to the spatial and time coordinates (finite difference method with approximation O(h^2^) and the Runge–Kutta type methods). Numerical results showed that, to achieve reliable conclusions, it is necessary to conduct a complex analysis and, owing to the proposed methodology, each signal should be studied separately.
2. The most negligible effect was observed when purple noise was added—beam vibrations remained periodic. On the other hand, beam vibrations were significantly influenced by pink noise. The degree of chaotization of the system essentially depends on the presence of low-frequency components in noise. The employment of the Morlet mother wavelets allowed to detect time evolution of frequencies during chaotic beam vibrations (Table 2 (α=0;1;2)).
3. It was found, illustrated and discussed that the Wolf (W), Kantz (K) and Rosenstein (R) methods may yield significantly different values of Lyapunov exponents for the same signal. On the other hand, all the above-mentioned methods exhibited good correlation when used to study different colored noises.
4. The obtained results indicate a need to employ a more complex study by using qualitatively different numerical approaches to obtain reliable/true chaotic vibrations.

It can be concluded that, to obtain the most reliable results, it is necessary to consider a mechanical system (flexible beam) as a system with an infinite number of freedom degrees. One should investigate the convergence of the solution when reducing the partial differential equations to the Cauchy problem by the second-order finite element method O(*h*^2^). The number of the beam length should be maximized (in this work, from n = 50 to *n* = 400 partitions were considered). The Cauchy problem can be solved by several other methods, such as Runge–Kutta method. For every problem, the largest Lyapunov exponents need to be investigated in several ways (for instance, by Wolf, Kantz, and Rosenstein methods). Finally, the presented complex and diverse research study guaranteed the reliability of the obtained results. 

## Figures and Tables

**Table 1:** Dynamical beam characteristics for *n* = 120; 320; 360; 400.

	Power Spectrum	Phase Portrait	Wavelet Spectrum
*N* = 120	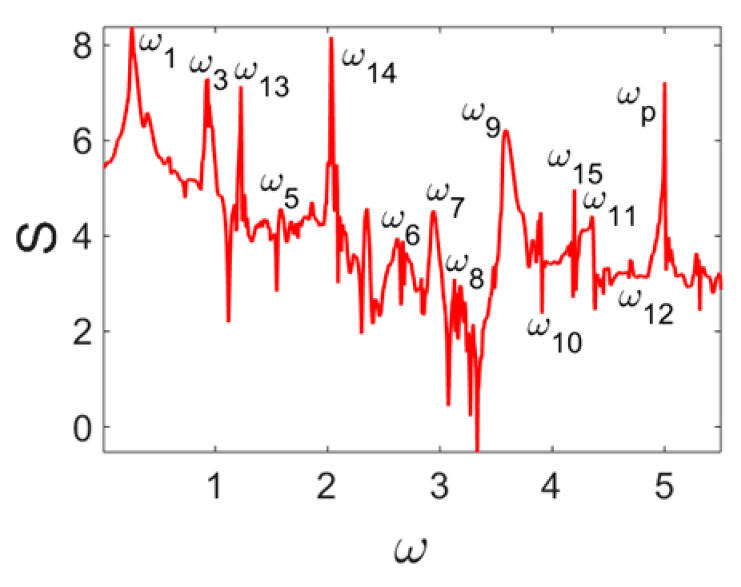	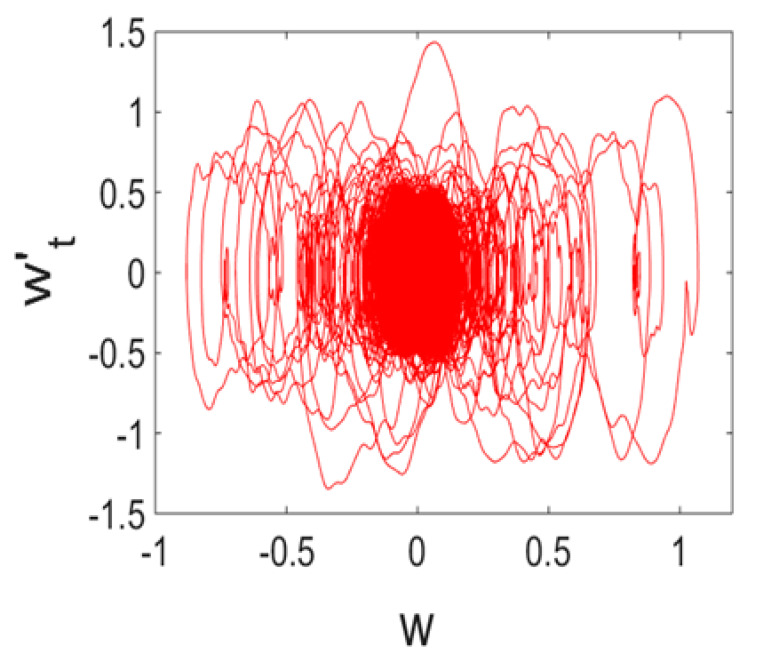	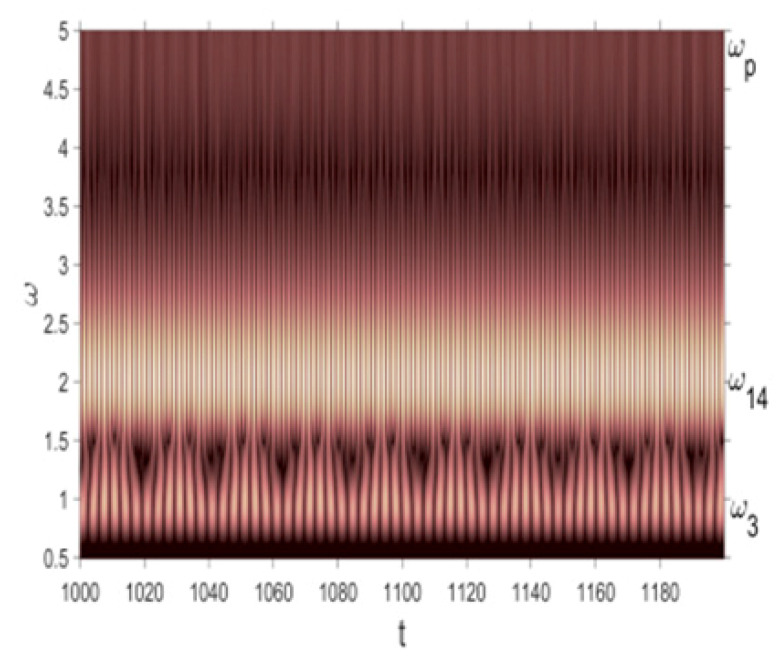
*N* = 320	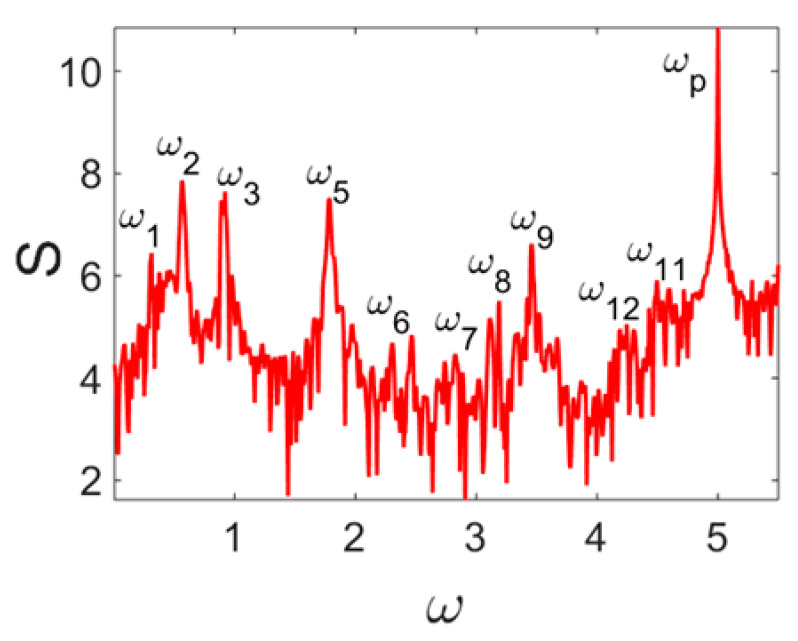	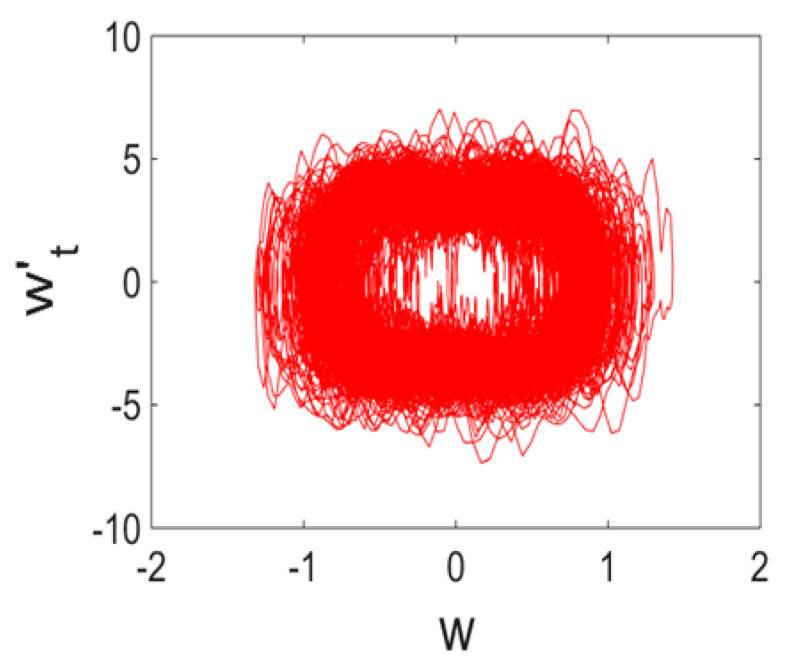	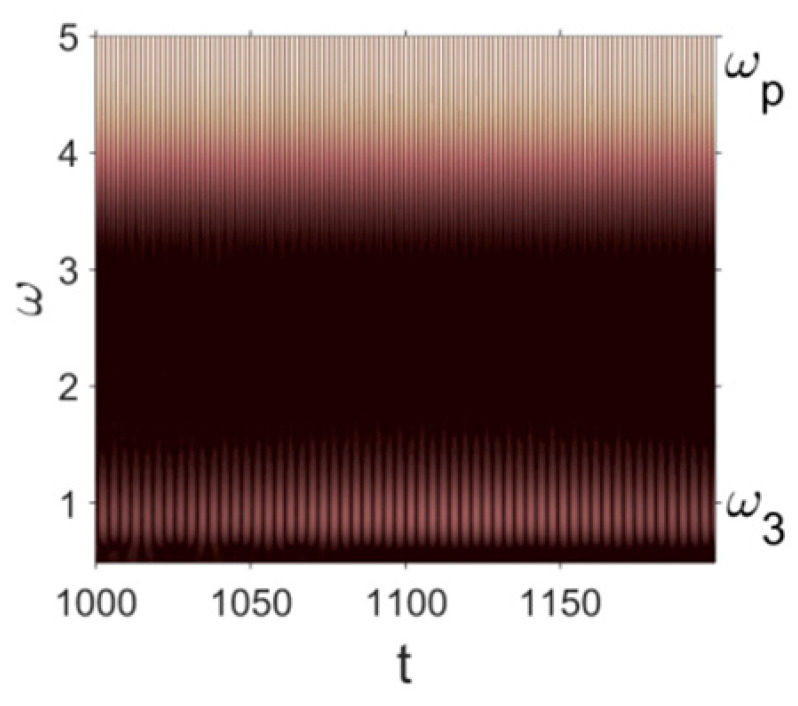
*N* = 360, 400	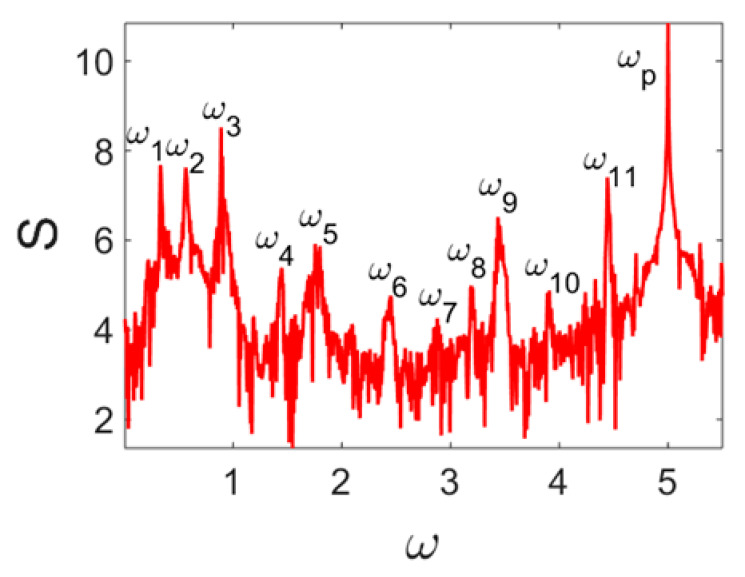	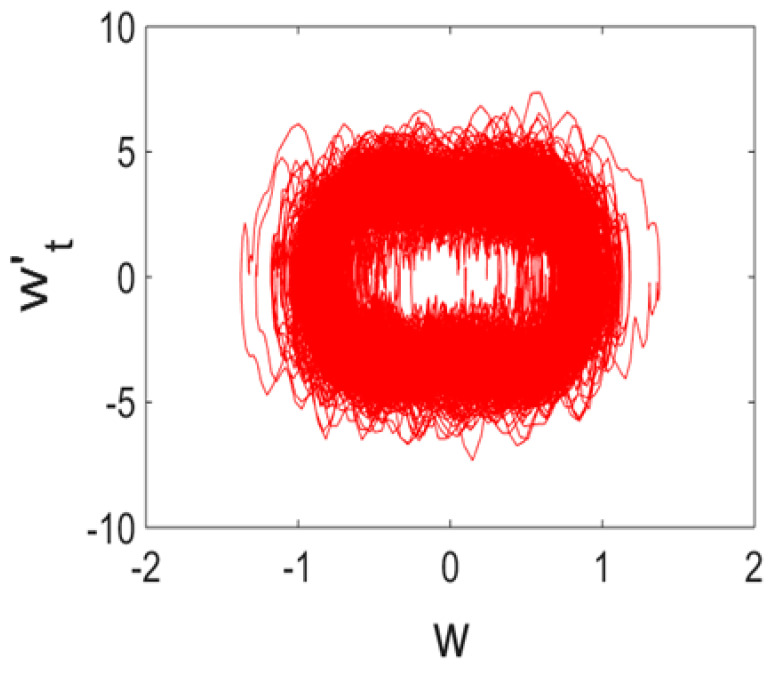	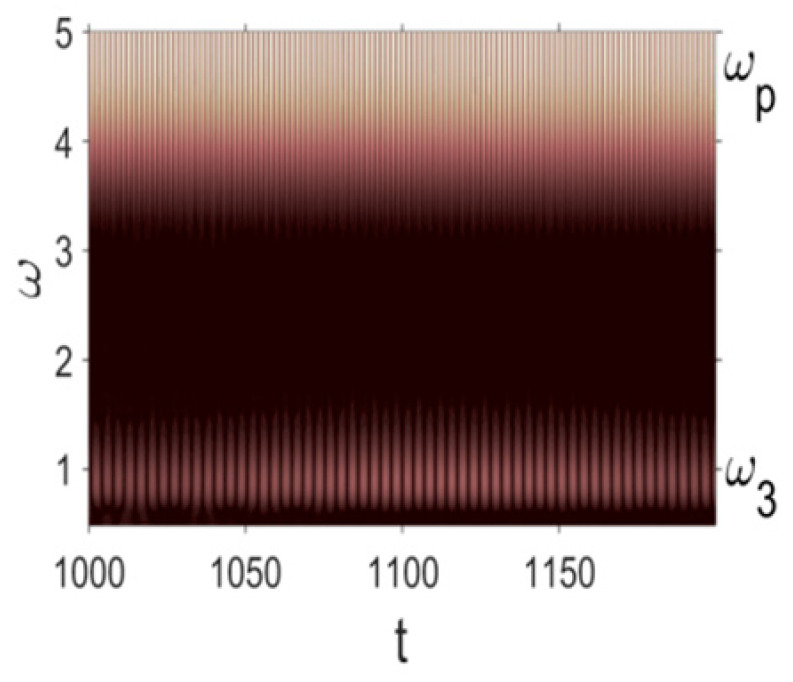

**Table 2:** LLEs (the largest Lyapunov exponents) computed by the Wolf, Rosenstein and Kantz methods.

*λ*	*q*	*n*	Wolf	Rosenstein	Kantz
50	10000	160	0.10078	0.11334	0.04127
50	10000	200	0.07357	0.07359	0.02888
50	10000	240	0.09443	0.08599	0.03110
50	10000	360; 400	0.09885	0.07995	0.03190

**Table 3:** Characteristics of the beam vibrations (*Cn* = 5000, *ω*_0_ = 1.0853, *q*_0_ = 5000).

Noise	Morlet Wavelet	Fourier Spectrum	*w*(*t*)	2D Phase Portrait	Poincaré Map
α=−2 purple	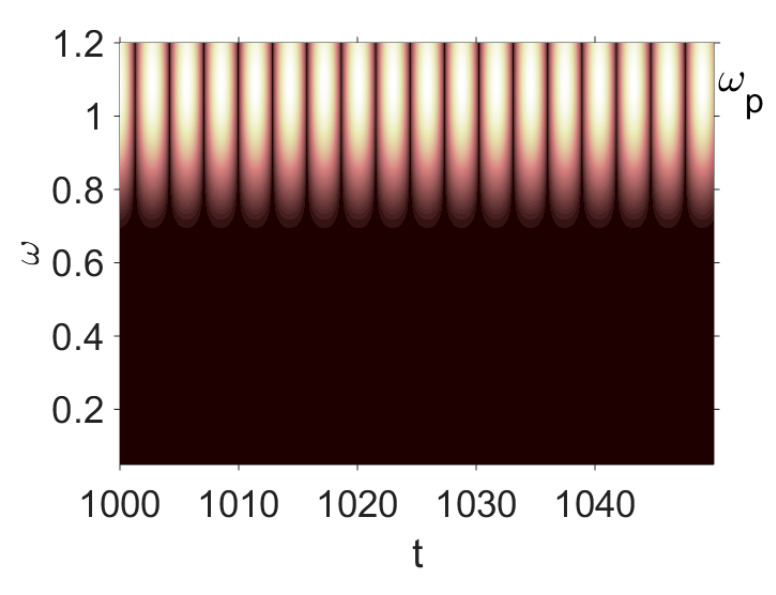	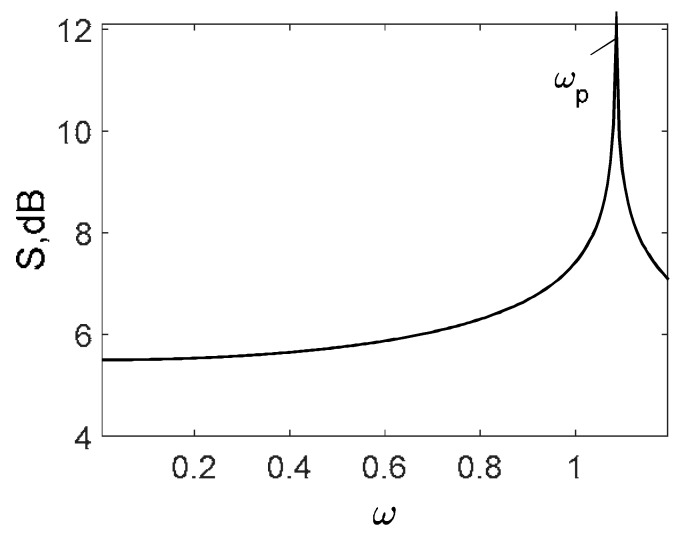	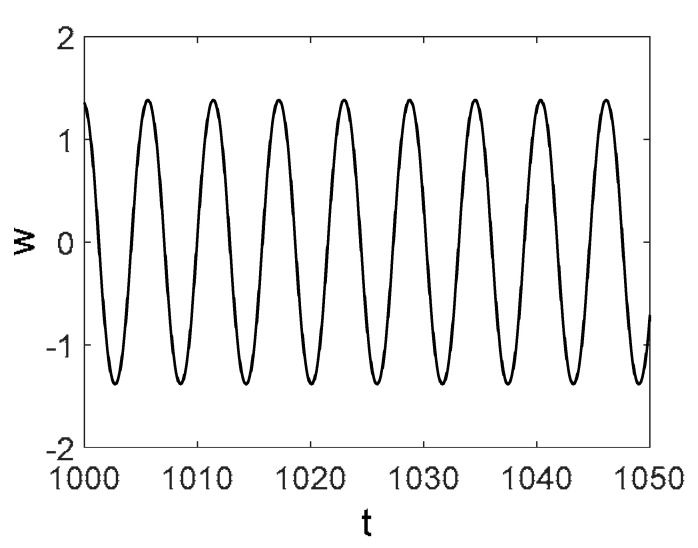	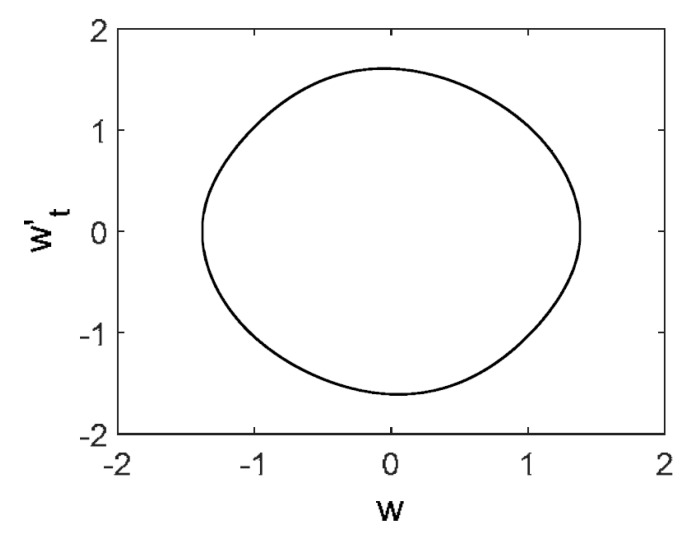	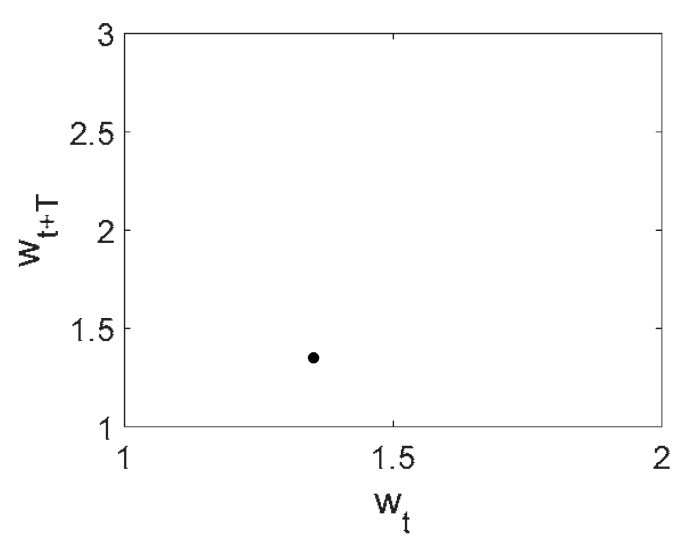
α=−1 blue	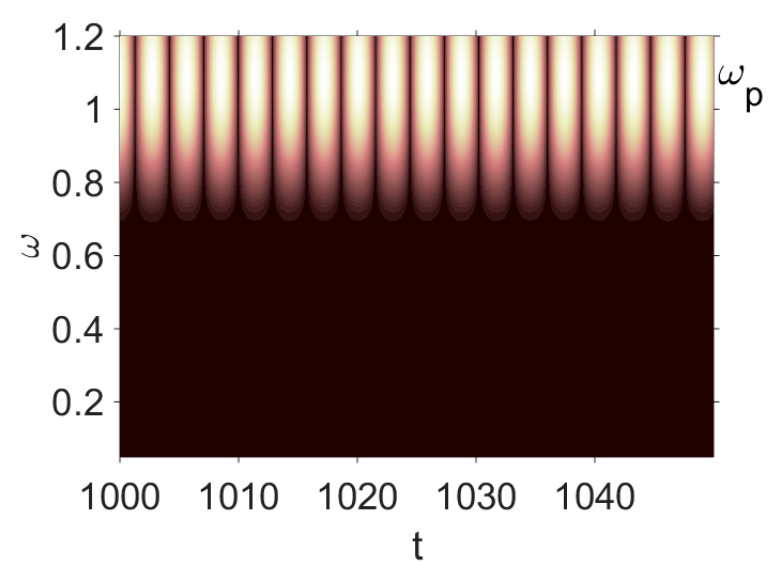	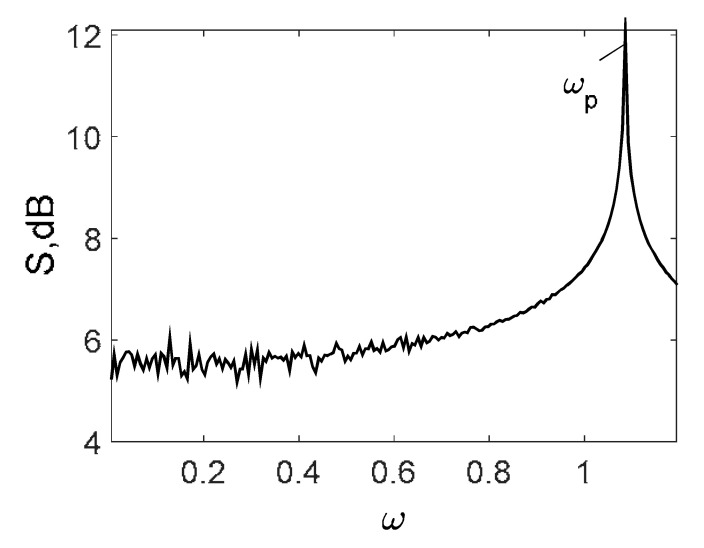	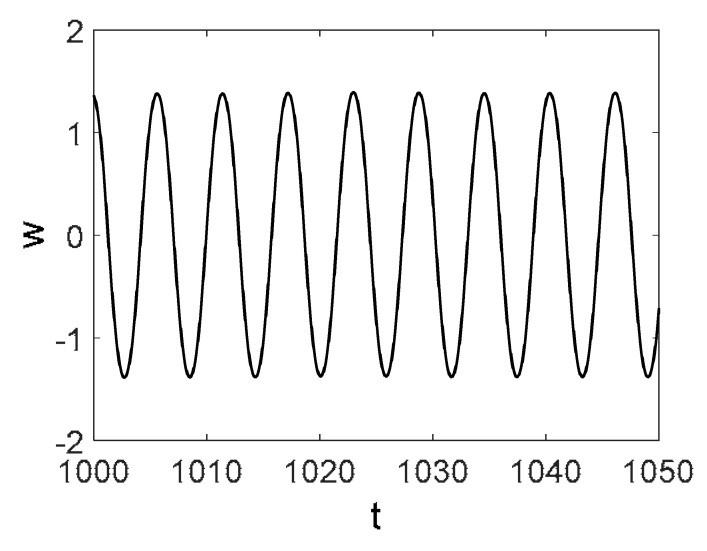	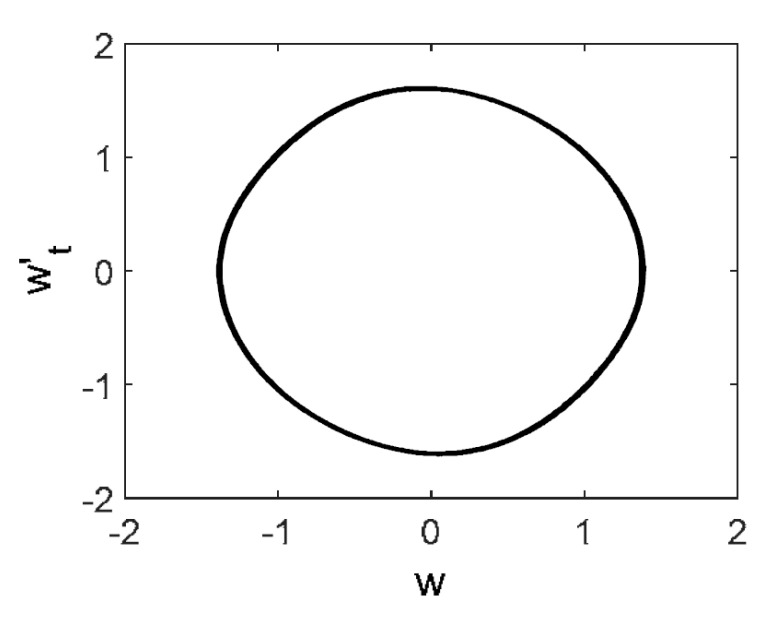	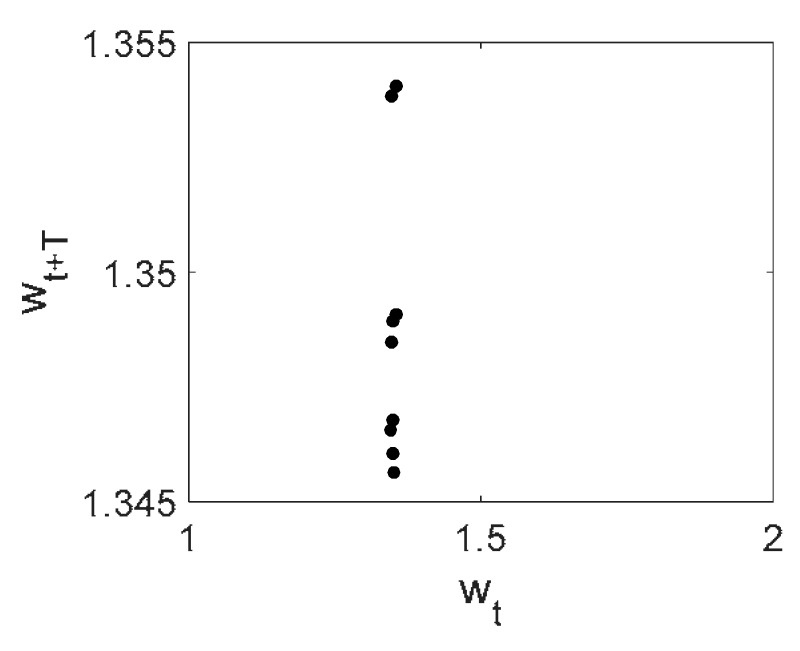
α=0 white	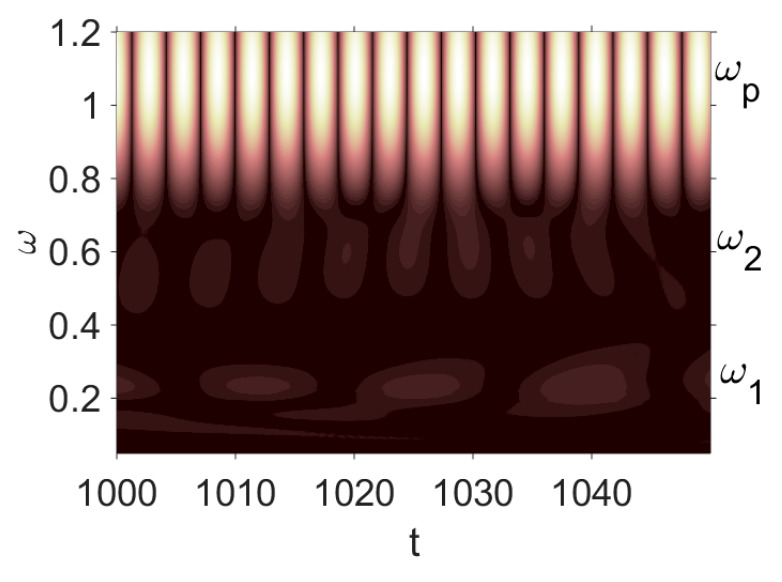	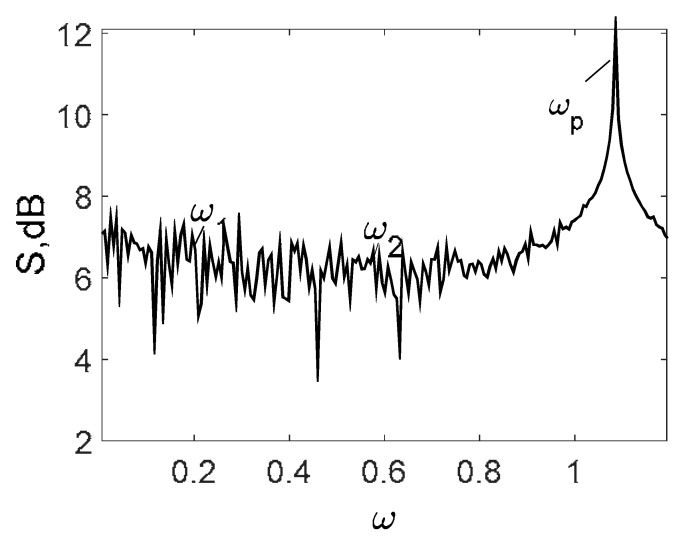	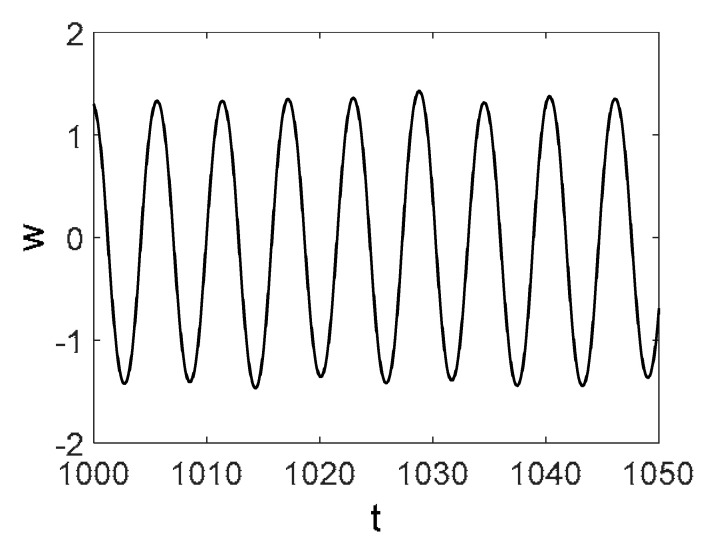	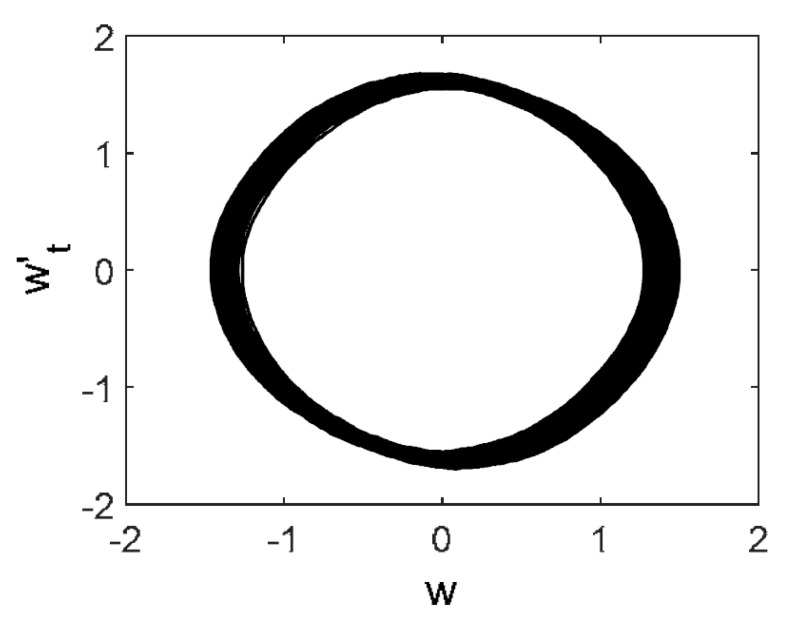	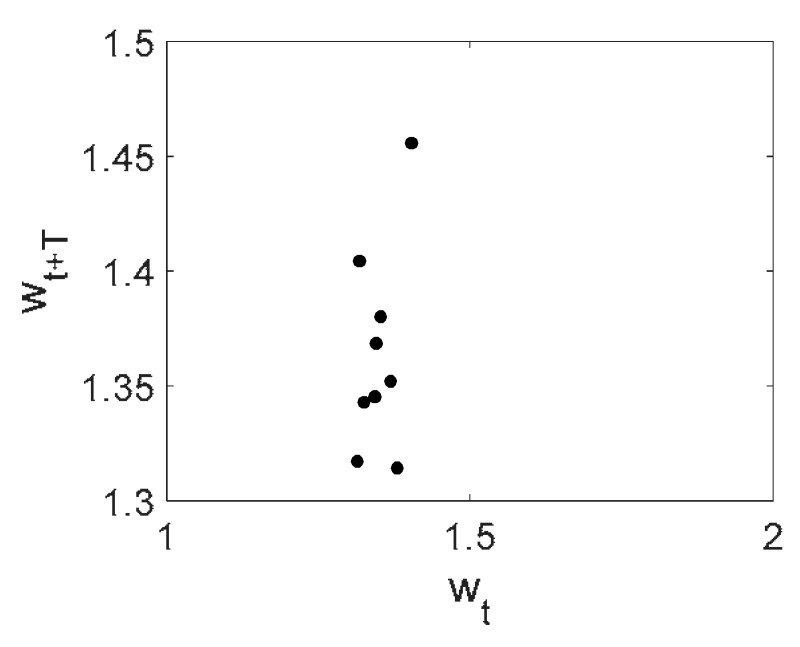
α=1 pink	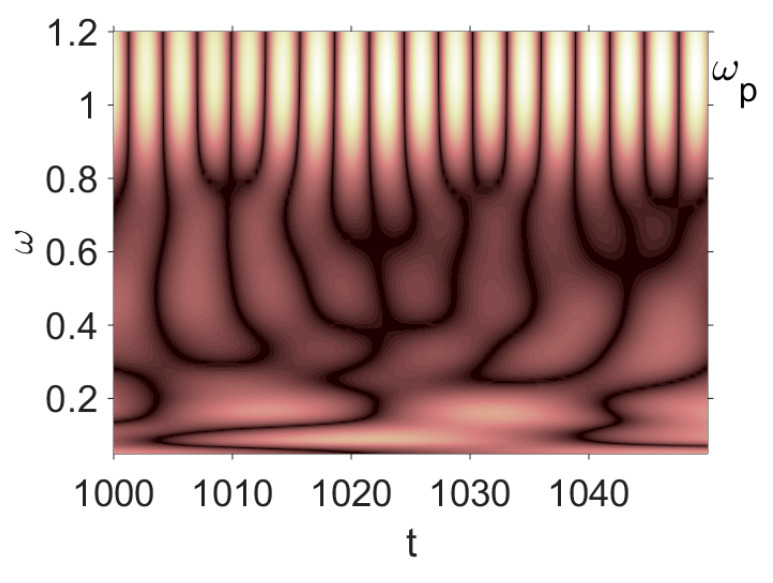	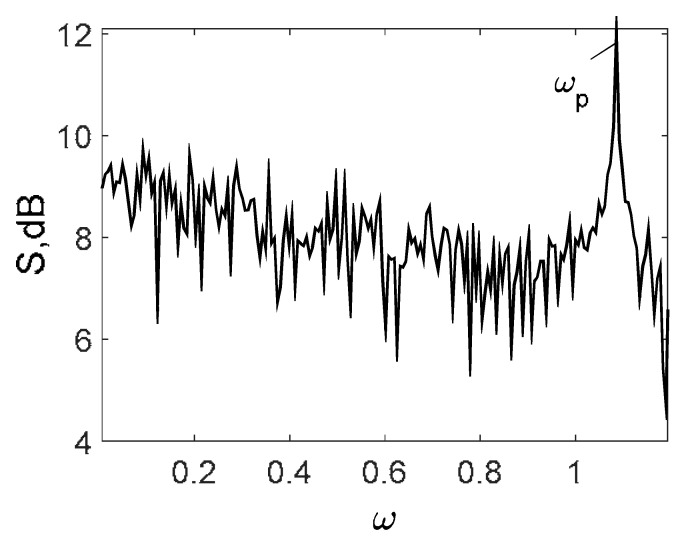	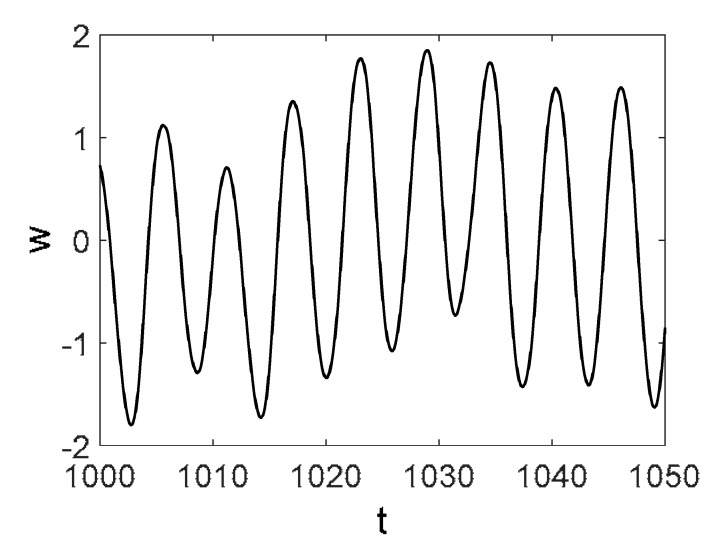	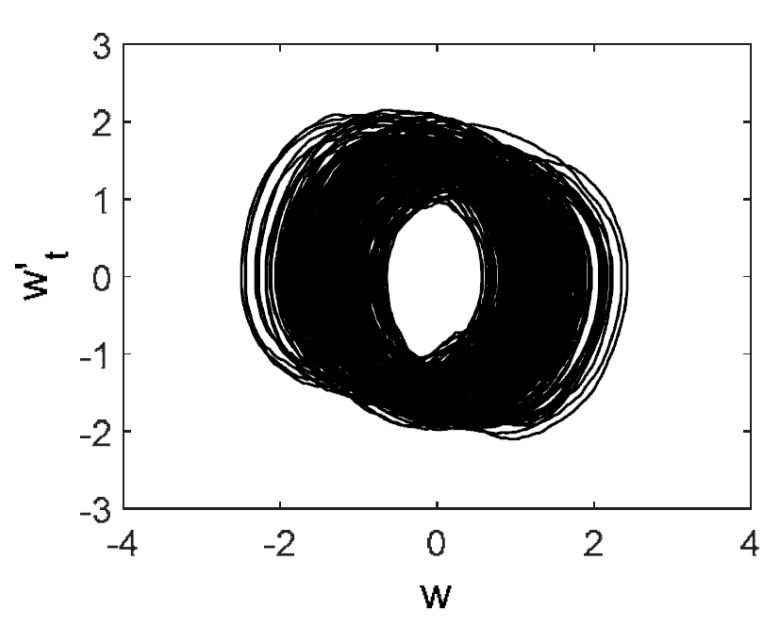	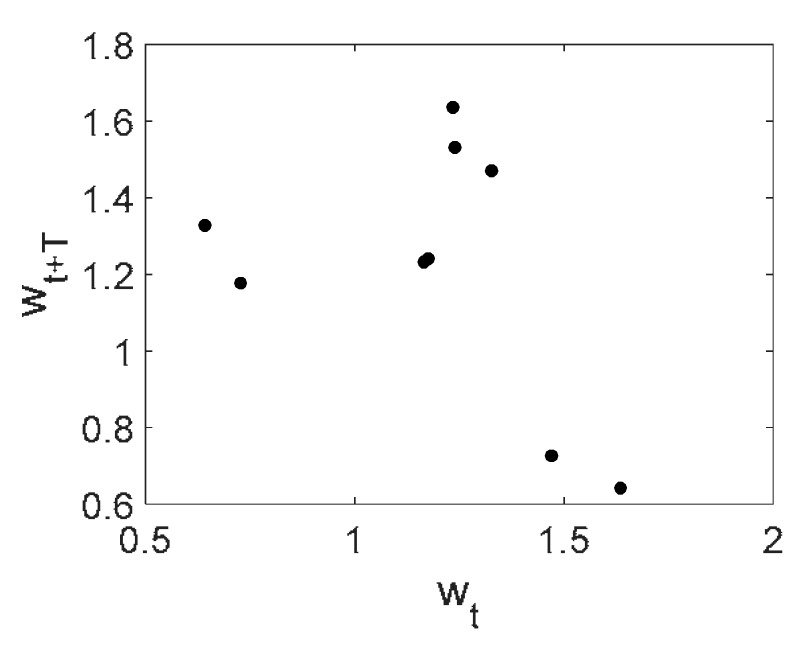
α=2 brown	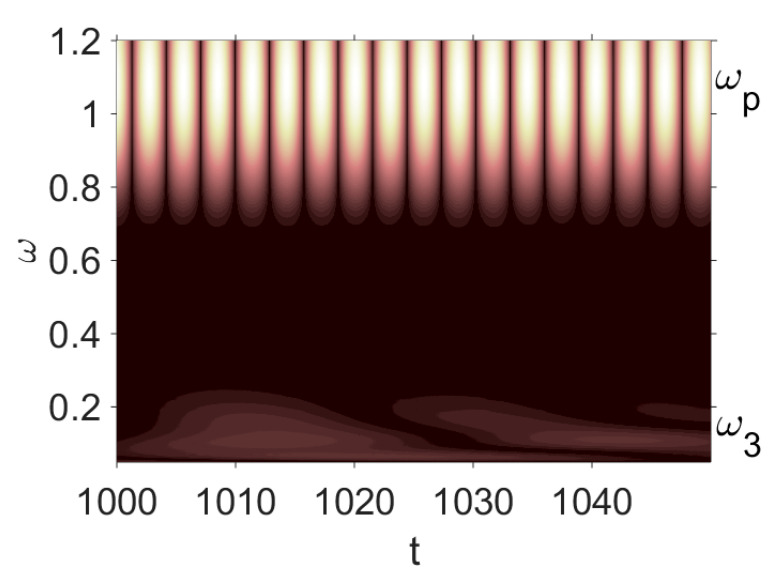	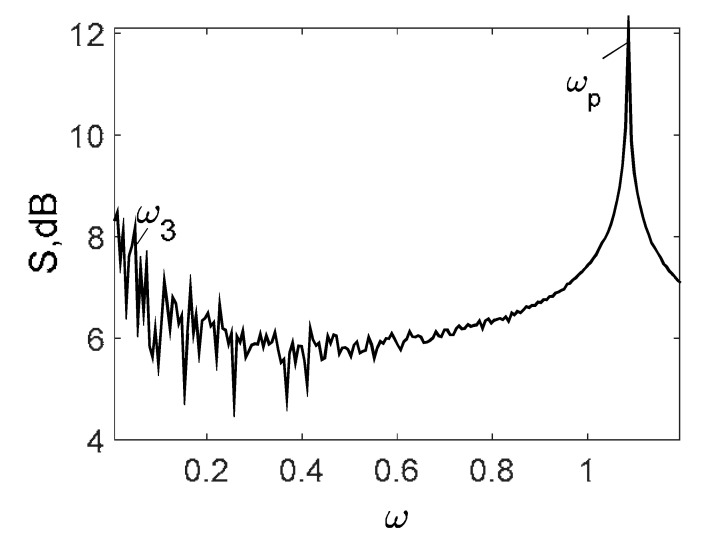	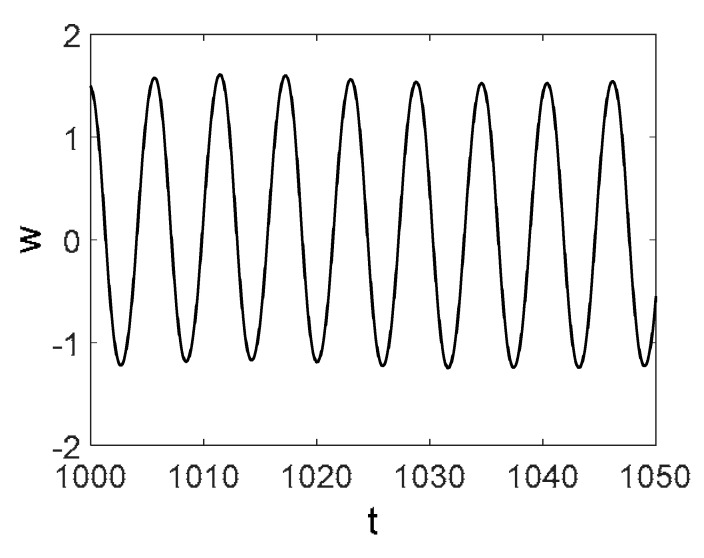	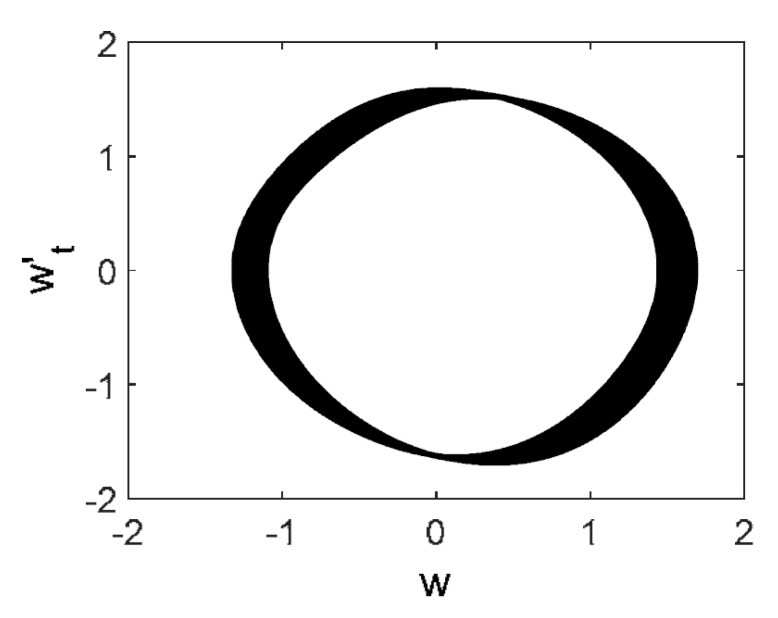	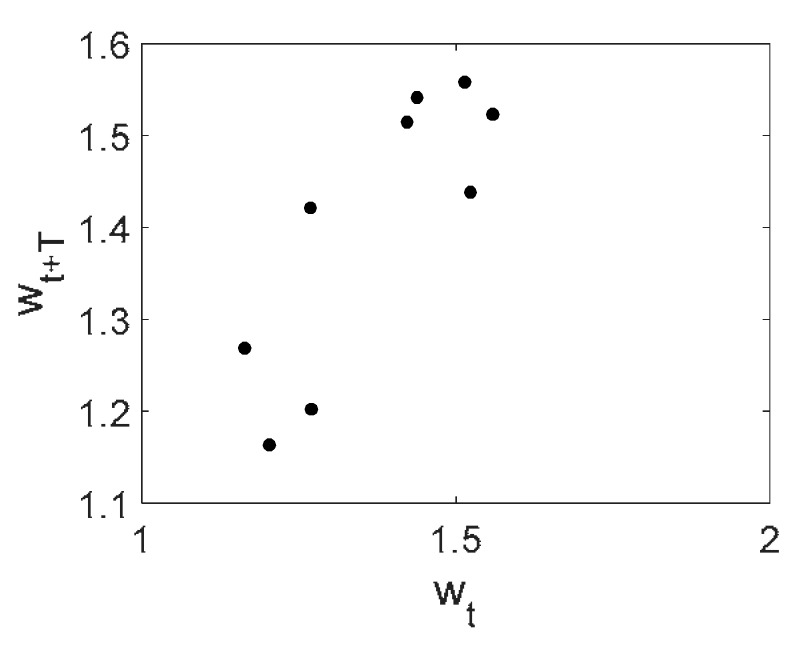

**Table 4:** Dependence of the average divergence of the neighborhood trajectories versus the considered evolution time *τ*.

Kantz	Rosenstein	Wolf
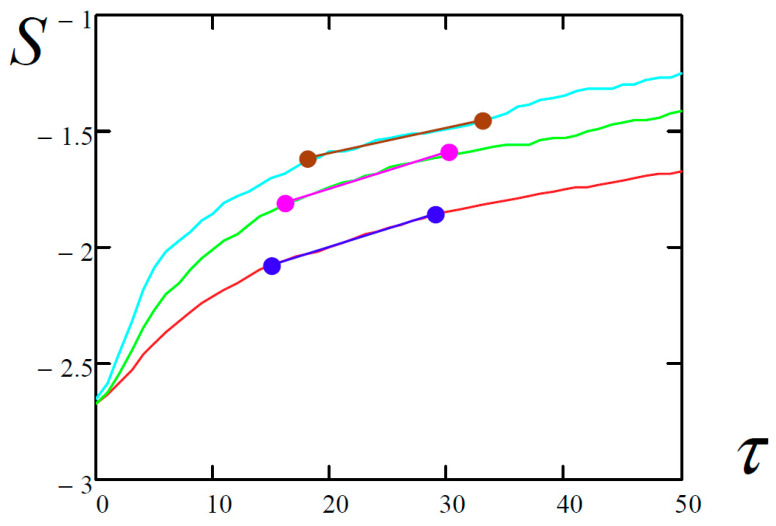	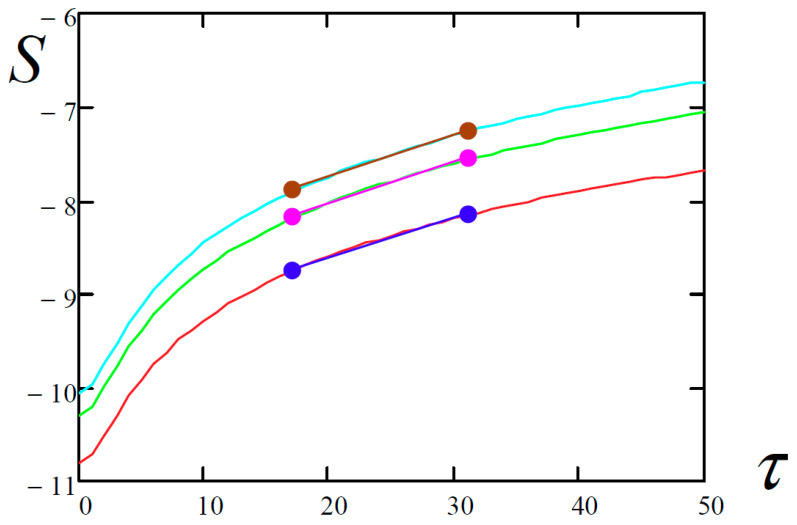	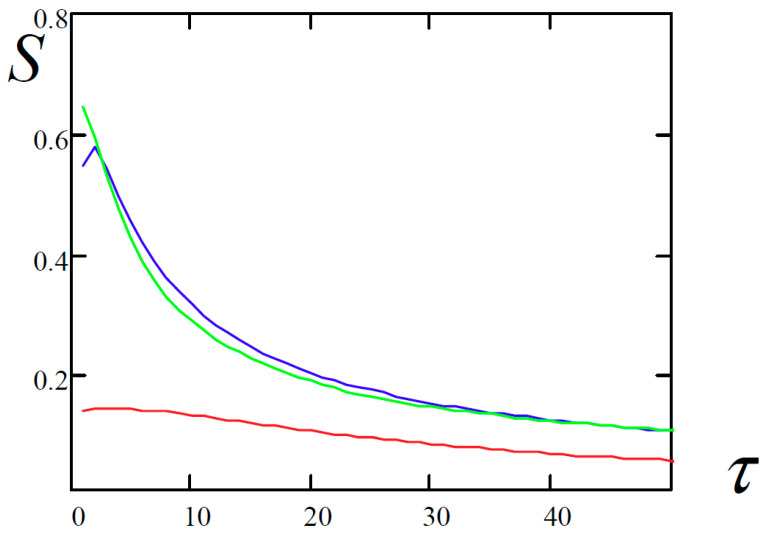

**Table 5:** LLEs for the investigated solutions (W/K/R state for the Wolf/Kantz/Rosenstein method, respectively).

α=−2	α=−1	α=0	α=1	α=2
W: 0.0 K: 0.0 R: 0.0	W: 0.0 K: 2⋅10^−2^ R: 6⋅10^−2^	W: 0.0 K: 3⋅10^−2^ R: 6⋅10^−2^	W: 0.0 K: 3⋅10^−2^ R: 7⋅10^−2^	W: 0.0 K: 2⋅10^−2^ R: 6⋅10^−2^
